# Targeting *Aspergillus fumigatus* Crf Transglycosylases With Neutralizing Antibody Is Relevant but Not Sufficient to Erase Fungal Burden in a Neutropenic Rat Model

**DOI:** 10.3389/fmicb.2019.00600

**Published:** 2019-03-26

**Authors:** David Chauvin, Michael Hust, Mark Schütte, Adélaïde Chesnay, Christelle Parent, Gustavo Marçal Schmidt Garcia Moreira, Javier Arroyo, Ana Belén Sanz, Martine Pugnière, Pierre Martineau, Jacques Chandenier, Nathalie Heuzé-Vourc’h, Guillaume Desoubeaux

**Affiliations:** ^1^INSERM, Centre d’Étude des Pathologies Respiratoires, U1100, Tours, France; ^2^Department Faculté de Médecine, Université de Tours, Tours, France; ^3^Institut für Biochemie, Biotechnologie und Bioinformatik, Technische Universität Braunschweig, Braunschweig, Germany; ^4^Service de Parasitologie – Mycologie – Médecine Tropicale, CHU de Tours, Tours, France; ^5^Departamento de Microbiología y Parasitología, Facultad de Farmacia, Universidad Complutense de Madrid, Instituto Ramón y Cajal de Investigación Sanitaria, Madrid, Spain; ^6^Institut de Recherche en Cancérologie de Montpellier, INSERM U1194, Université de Montpellier, Institut Régional du Cancer de Montpellier, Montpellier, France

**Keywords:** *Aspergillus*, fungi, invasive pulmonary aspergillosis, cell wall, Crf1, Crh5, glycosyltransferase, monoclonal antibody

## Abstract

*Aspergillus fumigatus* is an airborne opportunistic fungal pathogen responsible for severe infections. Among them, invasive pulmonary aspergillosis has become a major concern as mortality rates exceed 50% in immunocompromised hosts. In parallel, allergic bronchopulmonary aspergillosis frequently encountered in cystic fibrosis patients, is also a comorbidity factor. Current treatments suffer from high toxicity which prevents their use in weakened subjects, resulting in impaired prognostic. Because of their low toxicity and high specificity, anti-infectious therapeutic antibodies could be a new alternative to conventional therapeutics. In this study, we investigated the potential of *Chitin Ring Formation* cell wall transglycosylases of *A. fumigatus* to be therapeutic targets for therapeutic antibodies. We demonstrated that the Crf target was highly conserved, regardless of the pathophysiological context; whereas the *CRF1* gene was found to be 100% conserved in 92% of the isolates studied, Crf proteins were expressed in 98% of the strains. In addition, we highlighted the role of Crf proteins in fungal growth, using a deletion mutant for *CRF1* gene, for which a growth decrease of 23.6% was observed after 48 h. It was demonstrated that anti-Crf antibodies neutralized the enzymatic activity of recombinant Crf protein, and delayed fungal growth by 12.3% *in vitro* when added to spores. In a neutropenic rat model of invasive pulmonary aspergillosis, anti-Crf antibodies elicited a significant recruitment of neutrophils, macrophages and T CD4 lymphocytes but it was not correlated with a decrease of fungal burden in lungs and improvement in survival. Overall, our study highlighted the potential relevance of targeting Crf cell wall protein (CWP) with therapeutic antibodies.

## Introduction

Nowadays, medical practice is increasingly confronted with infectious pathology issues. In such a context, fungal diseases, from *Candida* sepsis in intensive care unit context, to airway mycoses which are the underlying cause of many cases of severe asthma and sinusitis, are a major concern (Porter et al., 2014; Jacobs et al., 2015). Invasive pulmonary aspergillosis (IPA) and allergic bronchopulmonary aspergillosis (ABPA) are both airborne diseases caused by ubiquitous molds of the *Aspergillus* genus, and primarily by *A.* *fumigatus* (Desoubeaux et al., 2014). IPA is responsible for high mortality rates ranging from 28.5 to 55% in immunocompromised hosts (Bitar et al., 2014; Taccone et al., 2015), following hematopoietic stem cell transplantation, solid organ transplantation or anticancer chemotherapy (Desoubeaux et al., 2014). Encountered in about 1.5% of patients with asthma (Latgé, 1999), ABPA is also encountered in 1–15% of patients suffering from cystic fibrosis (CF) (Stevens et al., 2003), for which massive colonization and subsequent hypersensitivity to *Aspergillus* spores are comorbidity factors, especially in cases of lung transplantation.

Together with an incomplete understanding of aspergillosis pathophysiology, the high fatality rate is partly caused by the lack of efficient antifungal therapies and the difficulty of establishing a reliable diagnosis. Azole- or polyene-based treatments suffer from numerous toxic side effects in liver, kidney or blood, which can prevent their use, and especially so in weakened patients. In addition, a progression in acquired resistance to azole treatments has been reported recently (Howard et al., 2009). In contrast, the echinocandin class shows lower toxicity, but displays only a fungistatic activity against *Aspergillus* species. Therefore, echinocandins are rarely used in first line in cases of aspergillosis (Walsh et al., 2008).

In view of these considerations, anti-infectious therapeutic antibodies are emerging as a promising alternative to conventional treatments (Kuhn et al., 2016; Sécher et al., 2018). They present the advantage of being very specific, therefore limiting unpredicted side effects. Some of them have already reached the pharmaceutical market, e.g., against respiratory syncytial virus (palivizumab), anthrax toxin (raxibacumab and obiltoxaximab) or *Clostridium difficile* toxin (bezlotoxumab), and a large number are currently under research and development process (Wagner and Maynard, 2018). Among them, a few have been developed against fungi, mostly against yeasts, and provided quite encouraging results (Matthews et al., 1995; Chaturvedi et al., 2005, 2009; Bugli et al., 2013).

An antibody fragment (MS112-IIB1) directed against the Chitin ring formation 2 (Crf2) protein of *A. fumigatus* was initially developed for diagnosis purposes (Schütte et al., 2009). Encoded by *CRF1* gene, the Crf protein family contains three protein variants: Crf2 protein, amino acids (333 aa) long, Asp f9 protein (292 aa), first identified as an allergen using serum from *Aspergillus*-allergic patients (Crameri and Blaser, 1996), and Crf1 (395 aa, also named Crh5). Crf1 protein is the only member of the Crf family with a predicted glycosylphosphatidylinositol (GPI) anchor (Arroyo et al., 2007), and has been shown to induce a cross-protection between *A. fumigatus* and *C. albicans* infection (Stuehler et al., 2011). Classified into the glycoside hydrolase family 16 (CAZy database), Crf proteins (Crf1, Crf2, Asp f9) are actually orthologs of Crh proteins in *A. fumigatus* (Crh1, Crh2, Crh3, Crh4), in *C. albicans* (Crh11, Crh12, Utr2) and *Saccharomyces cerevisiae* (Crh1, Crh2, Crr1) yeasts, and share a highly conserved catalytic domain (Arroyo et al., 2016). In yeasts, Crh proteins have been characterized with transglycosylase activity involved in the linkage of chitin to β(1-3)glucans and β(1-6)glucans residues in the cell wall (Cabib, 2009). In contrast, little is known about the conservation and the role of Crf proteins in *A. fumigatus*, despite quite a high structural similarity with Crh. Although β(1-6)glucans are not present in *A. fumigatus*, Crf may be involved in the remodeling of the cell wall as well, and therefore in the growth and the virulence of the fungus.

In this study, we highlighted the high conservation rate of Crf proteins in clinical strains of *A. fumigatus* and their potential as a therapeutic target. We demonstrated that anti-Crf MS112-IIB1 antibody might be a new option in the treatment of *A. fumigatus*-associated diseases, through its ability to neutralize Crf proteins *in vitro*. Moreover, spraying this antibody in a neutropenic rat model of IPA induced a significant recruitment of immune cells *in vivo*, but did not statistically increase the overall survival.

## Materials and Methods

### Strains

#### Clinical Strains

Forty-nine *Aspergillus fumigatus* strains were recovered at the CHRU of Tours (France): one in 2005 (referred to as “Crf+,” registered in the WFCC-MIRCEN *World Data Centre for Microorganisms* – Marseille, France – under no. BRFM 1827), and 48 between September and December 2015. Strains were either isolated from respiratory samples of patients without development of any *Aspergillus* disease (*n* = 38), or affected by ABPA (*n* = 4), IPA (*n* = 3), aspergilloma (*n* = 1), *Aspergillus* bronchitis (*n* = 1), or from environmental contamination (*n* = 2).

#### Engineered Strains

Ku80 Wild Type (WT) and Ku80 Crf knock-out mutant (Δ*CRF1*) strains were gently given by Dr. Daan Van Aalten at the University of Dundee, United Kingdom (Fang et al., 2015). Their engineering was performed based on the methods described by D’Enfert (1996) and Da Silva Ferreira et al. (2006).

### Culture Conditions

All fungal strains were initially grown on Sabouraud gentamicin chloramphenicol agar plates (Thermo Fisher Scientific, Dardilly, France) at 35°C for 72 h. Spores were harvested using 0.05% (v/v) Triton X100 in phosphate-buffered saline (PBS), followed by two washes in PBS with centrifugation at 1,700 ×*g* for 10 min, and re-suspended in PBS. Depending on the different protocols, spore concentration was adjusted in RPMI liquid medium (RPMI 1640, MOPS, glucose) or PBS (animal protocols) after a measurement of the absorbance at 530 nm followed by counting in hemocytometer cell.

### Antibodies

#### Bio-Engineering of scFv and scFv-Fc

Anti-Crf2 antibody MS112-IIB1 was produced as scFv as previously described (Schütte et al., 2009). Production of scFv-Fc with a human IgG1 Fc part was performed using the vector pCSE2.6-hIgG1-Fc-XP as described before (Jäger et al., 2013).

#### Production of IgG1

Variable regions from anti-Crf2 antibody MS112-IIB1 scFv coding plasmid were amplified by PCR to introduce BbsI restriction sites at their extremities. These fragments were used to construct a vector expressing a full-length human IgG1 using Golden Gate Assembly (Engler et al., 2008). The recipient vector was designed as described in Li et al. (2007), using the backbone of pFUSE-hIgG1-Fc1 (Invivogen, Toulouse, France) (Supplementary Figure S1B). The plasmid contains both IgG1 genes assembled as a bicistronic construct under the control of the hEF1 promoter before the light chain (Kappa constant domain), followed by an Encephalomyocarditis virus internal ribosome entry site to mediate heavy chain translation. The plasmids obtained were replicated in *E. coli* One Shot Top10 F’ (Thermo Fisher Scientific, Villebon-sur-Yvette, France) according to the supplier recommendations. Transformed bacteria were amplified in liquid medium LB low salt with Zeocin (Invivogen). Plasmids were then purified using Nucleobond Xtra Maxi EF 10 kit (Macherey-Nagel, Hoerdt, France). A transfection of HEK293F (Thermo Fisher Scientific) suspension cells was performed with Lipofectamine (FreeStyle MAX Reagent, Thermo Fisher Scientific), and cells were cultured for five days in FreeStyle 293 Expression Medium (Thermo Fisher Scientific). The supernatant was collected, and antibodies were purified on HiTrap Protein G HP affinity column (GE Healthcare, Buc, France) with ÄKTA chromatography system.

#### Surface Plasmon Resonance

Surface plasmon resonance experiments were performed on a Biacore 3000 instrument at 25°C using HBS-EP buffer [10 mM Hepes (pH 7.4), 3 mM EDTA, 150 mM NaCl, and 0.005% (v/v) non-ionic surfactant P20] (GE Healthcare) as running buffer. Recombinant *Af*Crh5 and rCrf2 were immobilized on Fc3 and Fc2, respectively, of a CM5 sensor chip surface at 300-400 RU by amine coupling according to the manufacturer’s instructions (GE Healthcare). Increasing concentrations of scFv, scFv-Fc and IgG1 were injected 180 s at 50 μL/min on *Af*Crh5, rCrf2 and a control flow cell (without the protein) simultaneously. After following the dissociation for 400 s, surfaces were regenerated by a short pulse of HCl at 100 mM. All sensorgrams were corrected by double referencing method and data were globally fitted to a Langmuir 1:1 or bivalent model using the BIA evaluation version 4.1.1 software.

### Epitope Mapping

Epitope mapping of MS112-IIB1 was performed in peptide array using an amino-cellulose membrane representing the sequence of Crf2 in a series of peptides (15mers overlapping by 12 amino acid residues) (Frank, 2002). After a blocking step in PBS 2% (w/v) skimmed milk, 0.05% (v/v) Tween 20 for 1.5 h at room temperature (RT), 40 μg scFv-Fc antibody were incubated in 10 mL PBS 2% (w/v) skimmed milk on the Crf2 membrane for 2.5 h at RT. After washing with PBS, the bound antibodies were detected with a secondary goat anti-human IgG antibody alkaline phosphatase conjugate (Dianova, Hamburg, Germany) diluted to 1:5,000 and incubated at RT for 1.5 h. Following two successive washing steps in 0.05% (v/v) Tween 20 PBS and in citrate-buffered saline (CBS) pH 7.0, staining solution [10 mL CBS, 1M MgCl_2_, 40 μL BCIP (Applichem, Darmstadt, Germany), 60 μL MTT (Sigma-Aldrich, Taufkirchen, Germany)] was added. The membrane was washed in PBS prior to being scanned and analyzed.

Localization of identified epitopes was performed on the *Af*Crh5 3D structure (Protein Data Bank n°5NDL) with UCSF Chimera 1.12 software (Pettersen et al., 2004).

### Crf and Crf2 Recombinant Proteins

*Af*Crh5 recombinant protein was kindly provided by Dr. Daan Van Aalten at the University of Dundee, United Kingdom (Fang et al., 2015). rCrf2 recombinant protein was produced as previously described (Schütte et al., 2009).

### Rat Model of Invasive Pulmonary Aspergillosis

*In vivo* experiments were performed using a previously described rat model of IPA (Chandenier et al., 2009). Briefly, male Sprague-Dawley rats (Janvier Labs, Le Genest-Saint-Isle, France), 6–8 weeks old, 200–225 g, were acclimated in animal facilities eight days before the beginning of the protocol. At day-5 (day 0 being the date of the infectious challenge), all animals were immunocompromised intraperitoneally with 75 mg/kg cyclophosphamide (Baxter, Guyancourt, France), and their food was changed for a low-protein diet (Safe Diets, Augy, France). Five-hundred milligrams tetracycline *per* liter (Sigma-Aldrich, Saint Quentin Fallavier, France) and 300 mg/L paracetamol (Sanofi-Aventis, Montrouge, France) were added to their drinking water to avoid opportunistic infections and limit pain. A second cyclophosphamide administration of 60 mg/kg was performed at day-1 to maintain immunosuppression. According to the protocols, animals were challenged intra-tracheally at day 0 by aerosolization of 300 μL of a PBS suspension containing 10^5^ or 10^6^ *A. fumigatus* conidia with Microsprayer IA-1B^®^ (PennCentury, Philadelphia, PA, United States). Rats were then monitored until the end of the protocol. Regarding the deleterious clinical signs of aspergillosis, sacrifices were performed before the end of the protocols if the following criteria were met: loss of weight ≥ 20%, discomfort score 3 (scored from 1 to 6 on the basis of appearance changes, e.g., dirty nose, red-rimmed eyes, ruffled fur, extreme pallor; score 1, no discomfort; score 2, minor discomfort; score 3, poor discomfort; score 4, serious discomfort; score 5, severe discomfort; score 6, death), behavior changes (e.g., gasping, wheezing, prostration, instability) and reaction to stimuli (Morton and Griffiths, 1985). At the sacrifice, blood, broncho-alveolar lavage fluids and lungs were collected for subsequent analysis.

### Sequencing of the *CRF1* Gene

After culture on Sabouraud gentamicin chloramphenicol agar plate for 24 h at 35°C, 80 mg *A. fumigatus* hyphae were harvested. Mycelium was disrupted with 0.1 mm glass beads (Ozyme, Montigny-le-Bretonneux, France) using Cryolys/Precellys 24 (Bertin Instruments, Montigny-le-Bretonneux, France) system in 380 μL of extraction buffer supplied in QIAmp DNA Mini Kit (Qiagen, Courtaboeuf, France), using two 25 s grinding steps at 6,800 rpm. Subsequent steps were performed accordingly to the supplier recommendations.

After DNA recovery, an amplification PCR was carried out using forward primer CCCAGTAGACTCGAGCTAGC and reverse primer CCCGATGCCGAAATGTATAG (Eurogentec, Angers, France) specific for *CRF1* gene, with an initial denaturation of 3 min at 94°C, followed by 35 cycles (30 s at 94°C, 30 s at 55°C, 45 s at 72°C), and a final elongation step of 15 min at 72°C. Sequencing was performed using BigDye Terminator v1.1 Cycle Sequencing Kit (Thermo Fisher Scientific), with in addition of the primers cited above, the following supplementary primers: CTTCCTTGACAAAACGCTCC, CCTGGCACAGGTGTTGTTAG, ACGTCAAGTCCGTCCGTATC, and AGCTAGAGCCAGAGCCAGAG. Sanger capillary electrophoresis was realized with 3130xl Genetic Analyzer (Applied Biosystems, Villebon-sur-Yvette, France), as recommended by the supplier instructions. Data were analyzed using CodonCode Aligner 6.0.2 (CodonCode Corporation, Centerville, MA, United States) software.

### Analysis of the Expression Levels of *CRF1* RNA Variants

#### Quantification of RNA Levels *in vitro*

*A. fumigatus* spores were seeded at 2.37 × 10^5^ *per* well on 6-well plate in 4 mL of RPMI medium, and were incubated at 35°C for 24 h. After removing the medium, the mycelium was harvested adding 700 μL TRIzol Reagent (Thermo Fisher Scientific) and grinded with 0.1 mm glass beads (Ozyme), using Cryolys/Precellys 24 (Bertin Instruments) system, for two 25 s cycles at 6,500 rpm. The following RNA isolation steps were performed with RNeasy Plant Mini Kit (Qiagen) according to the supplier’s instructions. Recovered RNA was quantified and its integrity was analyzed by RNA6000 Nano kit (Agilent, Courtaboeuf, France). Reverse transcription was performed with SuperScript II Reverse Transcriptase kit (Thermo Fisher Scientific) and cDNA was quantified by RT-qPCR. Specific primers of transcripts Crf1 (Forward: GGAACCTACTACCACCGGC, Reverse: GTGACCGAGCCCTTGATGG), Crf2 (Forward: CTACTCGGACAACTCTGGCTC, Reverse: ACGGCAGCGGTAGGTTCC), Asp f9 (Forward: CAAGTCCGTCCGTATCGAGA, Reverse: TTTGTTTACGAGGTAGAGCTGG), and housekeeping gene transcripts gpdA (glutaraldehyde 3-phosphate dehydrogenase) (Forward: CACCGTCCACTCCTACACC, Reverse: GCTTGCCGTTGAGAGAAGG) and TUB1 (β-tubulin) (Forward: CTTCCAGGTCACCCACTCTC, Reverse: CTGGTGAACGGAGAGGGTAG) were used. Amplification was carried out using the Taqman method, with the following probes: Crf1: FAM-CAGCAGCAACACCGGCTCTG-BHQ1, Crf2: FAM-ACCTCCACCCTGGCCACTTC-BHQ1, Asp f9: FAM-ACCTCCTCCACCACCAGCAC-BHQ1, gpdA: HEX-TGGTCGTACTGCTGCCCAGAA-BHQ1 and TUB1: CY5-CCGACCGTATGATGGCGACCTT-BHQ2. Platinum Quantitative PCR SuperMix-UDG (Invitrogen) was used to perform the amplification on LightCycler 480 (Roche, Boulogne-Billancourt, France) with the following program: decontamination 2′ at 50°C, initial denaturation 10′ at 95°C, first amplification cycle 15″ at 95°C and 1′ at 58°C, second amplification cycle 15″ at 95°C and 1′ at 59°C, third amplification cycle 15″ at 95°C and 1′ at 60°C, fourth amplification cycle 15″ at 95°C and 1′ at 61°C, fifth amplification cycle 15″ at 95°C and 1′ at 62°C, followed by 50 cycles 15″ at 95°C and 1′ at 63°C. The cycle threshold values of each Crf1, Crf2 or Asp f9 transcripts were then compared to the geometric mean of the cycle threshold values of both housekeeping genes, allowing the calculation of a ratio and the assessment of their relative expression.

#### Quantification of RNA Levels *in vivo*

Lung tissues from rats infected with 10^5^ conidia of Crf+ strain were sliced and collected in RNAlater solution (Sigma-Aldrich), prior to a grinding with GentleMACS (Miltenyi Biotec, Paris, France) system. After an incubation for 24 h at 4°C, 25 mg of tissue were harvested and centrifuged at 3,000 ×*g* for 5 min. Tissue was then re-suspended in 700 μL TRIzol Reagent (Thermo Fisher Scientific) and RNA extraction and quantitation steps were performed as previously described for *in vitro* cultivated strains.

### Analysis of the Expression of Crf Proteins

#### *In vitro* Expression by Immunofluorescence

Crf+ strain conidia were seeded at 1.05 × 10^4^ spores *per* well in Lab-Tek 8-well chamber slide (Nalge Nunc International, Villebon-sur-Yvette, France) in 300 μL RMPI medium, and incubated at 35°C for 8, 10, 14, or 24 h. At the end of the incubation, for each well, culture medium was removed and 300 μL ice cold methanol were added for 10 min. A blocking step was performed using 300 μL of a PBS solution containing 3% (w/v) BSA (Sigma-Aldrich), 2% (w/v) skimmed milk powder (Régilait, Macon, France) and 0.05% (v/v) Tween 20 (Thermo Fisher Scientific) for 10 min. Two hundred microliters of either scFv-Fc anti-Crf MS112-IIB1 antibody or isotype control diluted at 2 μg/mL in the previous solution without Tween 20 were incubated during 1 h at RT. Secondary antibody (anti-human IgG Alexa Fluor 488, Thermo Fisher Scientific) was added at 1:200 for 1 h at RT. Cell nuclei were stained with 1:1,000 Hoechst (Interchim, Montluçon, France) solution, before observing the slide using confocal microscopy (Olympus FV500).

#### *In vitro* and *in vivo* Expression by Western Blot

*A. fumigatus* spores were seeded in 6-well plates at a density of 2.37 × 10^5^ spores *per* well in 4 mL of RPMI medium for 24 h at 35°C. CWPs extraction was adapted from Pitarch et al. (2008) protocol. Briefly, culture medium was removed, and wells were washed with 10 mM Tris-HCl pH 7.4, 5 mM EDTA. The fungus was then harvested and suspended in 1 mL of the previous solution before adding final 2 mM PMSF and 10 μL inhibitor cocktail (Sigma-Aldrich). The suspension was grinded with 0.1 mm glass beads (Ozyme), using Cryolys/Precellys 24 (Bertin Instruments) system, for two 25-s cycles at 6,800 rpm. The cell wall suspension recovered was centrifuged at 1,500 ×*g* for 10 min and weighed. Three micrograms of cell wall extract were washed with 5 mL of 50 mM Tris-HCl pH 7.5 solution with 1 mM PMSF. After centrifugation at 1,500 ×*g* for 10 min, cell wall extracts were incubated at 37°C for 22 h in 1 mL of the previous solution containing 1 mM PMSF, 10 mM DTT and 1000 U lyticase (Sigma-Aldrich), under agitation. The suspension was then centrifuged at 12,000 ×*g* for 10 min and the supernatant was collected. Proteins were precipitated adding 1:10 TCA (Thermo Fisher Scientific) 100% followed by an incubation in ice for 30 min. After a 10,000 ×*g* centrifugation for 15 min, pellets were washed twice in cold acetone and dried. All samples were frozen at -80°C until the Western Blot analysis was performed.

After collection, human BALF samples were centrifuged at 3,000 ×*g* for 10 min. Pellets were re-suspended in 1 mL 10 mM Tris-HCl pH 7.4, 5 mM EDTA and grinded, as described above.

Lungs of rats infected with 10^6^ conidia were sliced in 25 mg portions, prior to being re-suspended in 1 mL 10 mM Tris-HCl pH 7.4, 5 mM EDTA and grinded, as described previously.

Pellets containing precipitated CWPs were re-suspended in 20 μL of 1X NuPAGE LDS sample buffer (Thermo Fisher Scientific). Samples were dropped on NuPAGE 10–20% Tris-Glycine (Thermo Fisher Scientific) electrophoresis gel, under non-denaturing and non-reductive conditions. Respectively 0.02 μg and 0.07 μg of recombinant *Af*Crh5 and rCrf2 proteins were diluted in the same buffer and deposed on the gel. Migration was performed in Tris-Glycine-SDS buffer, and proteins were transferred from the gel to a PVDF membrane by electrophoretic liquid transfer in Tris-Glycine buffer. Following a saturation step in Tris-buffered saline 0.1% (v/v) Tween 20 with 5% (w/v) of skimmed milk powder (Régilait), primary antibody (scFv-Fc MS112-IIB1) at 2 μg/mL was incubated on the membrane overnight at 4°C. Anti-human IgG HRP-coupled secondary antibody (Jackson ImmunoResearch, West Grove, PA, United States) was then incubated for 1.5 h at RT. The membrane was developed using SuperSignal West Pico Chemiluminescent Substrate (Thermo Fisher Scientific), with a detection on photographic film.

#### *In vivo* Expression by Immunohistochemistry

Lungs of rats infected with Crf+ or Crf- strains were collected in 4% buffered formaldehyde (VWR, Fontenay-sous-Bois, France) before changing to ethanol 70% 24 h later. Lungs were embedded in paraffin before preparing 4 μm slides. Slides were thereafter rehydrated in successive baths of xylene and ethanol. Following an antigen retrieval step in Tris-EDTA pH 9.0 with 0.05% (v/v) Tween 20, an endogenous peroxidase inactivation step in methanol 5% (v/v) H_2_O_2_ (Sigma-Aldrich) was performed. Blocking was performed with 5% (v/v) normal goat serum (Sigma-Aldrich) and 2% (w/v) bovine serum albumin (BSA) (Sigma-Aldrich) during 1.5 h, before adding primary anti-Crf antibody at 2 μg/mL for 1.5 h. A negative control was carried out in parallel, incubating primary antibody with recombinant rCrf2 protein. Subsequent steps with secondary biotinylated antibody and HRP-avidin addition were achieved using Vectastain ABC kit Human IgG (Vector Laboratories, Peterborough, United Kingdom), until revelation in DAB (Dako, Les Ulis, France) and counter-coloration with Gill’s hematoxylin. Slides were then dehydrated and scanned.

### Study of Crf Protein Neutralization *in vitro*

#### Assessment of Crf Deletion Impact on Fungal Growth

Ku80 WT and Ku80 Δ*CRF1* strains conidia were seeded at 3.5 × 10^4^ *per* well in a 96-well plate, in 200 μL RPMI medium. Fungal growth kinetics was studied over 48 h by measuring the 530 nm absorbance every 30 min (Infinite 200 Pro, Tecan, Lyon, France), at 12 different points *per* well, to overcome the heterogeneity due to the mycelium growth. Spectrophotometry method was validated using MTT method (not shown).

#### Neutralization of Crf Enzymatic Activity

The assay to measure transglycosylase activity of Crf protein was carried out as previously described (Mazan et al., 2013). Nine micrograms of recombinant *Af*Crh5 protein were pre-incubated with PBS, IgG1 trastuzumab anti-HER2 antibody (control), or IgG1 MS112-IIB1 antibody (anti-Crf) in a 2:1 molar ratio (antibody:*Af*Crh5) for 30 min at room temperature. A mixture containing 18 μM sulforhodamine-labeled oligosaccharide (laminaripentaose L5-SR or chitopentaose CH5-SR) as the acceptor, and carboxymethyl-chitin (0.1%) as a donor, in 50 mM citrate buffer pH 5.8, was then added to the reaction in a final volume of 150 μL. Transglycosylation was carried out at 37°C and aliquots of 20 μL were taken for each time point. The reaction was stopped by addition of 20 μL of 40% (v/v) formic acid. Aliquots of 5 μL from the stopped mixture were spotted in quadruplicates onto a filter paper and processed as previously described (Mazan et al., 2013). Enzymatic activity was then determined by a fluorescence measure (expressed in fluorescence units) in a FLUOStar Galaxi (BMG Labtech, Ortenberg, Germany) ELISA microplate reader equipped with a fluorescent detector and filters with an excitation wavelength of 540 ± 10 nm and emission wavelength of 570 ± 10 nm.

#### Neutralization of Fungal Growth

*A. fumigatus* spores were seeded at 3.5 × 10^4^ spores *per* well in a 96-well plate in 100 μL of RPMI. One hundred microliter, either of RPMI alone, IgG1 MS112-IIB1 antibody or control antibody (IgG1 trastuzumab anti-HER2) were then added to each well, on spores (0 h, no incubation), on germinating spores (after a 6 h-long incubation at 35°C) and on 24 h hyphae (after a 24 h-long incubation at 35°C). Final concentration was adjusted in RPMI at 0.5 μg/mL of antibody. Fungal growth was evaluated after a 48 h incubation at 35°C, *via* measurements of the absorbance at 530 nm at 12 different points *per* well.

### Study of Crf Protein Neutralization *in vivo*

#### Testing of IgG1 Antibody

Thirty rats were intra-tracheally challenged with 10^5^ *A. fumigatus* spores of Crf+ strain and treated extemporaneously with PBS (*n* = 6), trastuzumab IgG1 control antibody at 4 mg/kg (*n* = 12) or MS112-IIB1 IgG1 anti-Crf antibody at 4 mg/kg (*n* = 12). After 32 h, all animals were treated again with intra-tracheal administration of PBS (control group), 4 mg/kg trastuzumab (control antibody group) or 4 mg/kg MS112-IIB1 IgG1 (anti-Crf antibody group), respectively. Seventy-two hours after initial challenge, all rats were sacrificed and lungs were collected. Lung tissues were sliced and digested in 9 mL RPMI with 5% (v/v) fetal bovine serum (FBS) and 125 μg/mL liberase (Roche) for 30 min at 37°C, using GentleMACS (Miltenyi Biotec) system. Homogenates were divided in two for fungal load assessment and study of immune cells recruitment, and centrifuged at 400 ×*g* for 5 min at 4°C.

#### Assessment of Immune Cell Recruitment by Flow Cytometry

Homogenate pellets were re-suspended in 4 mL RPMI with 5% (v/v) FBS and 0.5 mg/mL DNAse (Roche) and incubated with shaking at 37°C for 30 min. Following a filtration (100 μm) step, homogenates were treated with red cells lysing solution (0.1 mM EDTA, 10 mM KHCO_3_, 150 mM NH_4_Cl) during 15 min. Samples were centrifuged at 400 ×*g*, 5 min at 4°C, re-suspended in 5 mL PBS 5% (v/v) FBS and filtered again (100 μm). After a final centrifugation step at 400 ×*g*, 5 min at 4°C, recovered cells were re-suspended in PBS with 5% (v/v) FBS and 2 mM EDTA (working buffer). For each rat, cells number was determined with MACS Quant flow cytometer (Miltenyi Biotec), and four wells of a 96-well round bottom plate were seeded with 500,000 cells. After centrifugation, 50 μL of working buffer with 0.5% (w/v) mouse anti-rat CD32 (BD Biosciences, Le Pont de Claix, France) were added to each well and incubated for 15 min. Four antibody mixes were prepared for the study of immune cell populations in flow cytometry. To each mix, 1:1,000 Live-dead stain (Thermo Fisher Scientific) and 0.1 μg mouse anti-CD45 APC-eFluor 780 (Thermo Fisher Scientific) were added. In the neutrophils, total macrophages, alveolar macrophages and interstitial macrophages mix, cells were stained with 0.1 μg mouse anti-rat CD11b V450 (BD Biosciences), 1 μg rat anti-rat Ly6G FITC (Abcam, Paris, France) and 0.2 μg mouse anti-rat CD172a Pe (BioLegend, London, United Kingdom). In T CD4, T CD8 lymphocytes and natural killers mix, cells were stained with 0.1 μg mouse anti-rat αβ T-cell PerCP (BD Biosciences), 0.25 μg mouse anti-rat CD4 FITC (BD Biosciences), 0.2 μg mouse anti-rat CD8a V450 (BD Biosciences) and 0.025 μg mouse anti-KLRB1 Pe (Thermo Fisher Scientific). In the B lymphocytes mix, cells were stained with 0.5 μg mouse anti-rat CD45R FITC (BD Biosciences) and 0.4 μg mouse anti-rat IgM Pe (BD Biosciences). In dendritic cells and activated dendritic cells mix, cells were stained with 0.2 μg mouse anti-rat CD11c AF647 (AbD Serotec, Kidlington, United Kingdom), 0.5 μg mouse anti-rat MHC Class II Pe (Bio-Techne, Lille, France) and 0.5 μg mouse anti-rat CD86 AF405 (Bio-Techne). All mixes were incubated at 4°C for 30 min. After centrifugation, cells were re-suspended in 200 μL working buffer except neutrophils/macrophages mix in which cell were permeabilized with BD Cytofix/cytoperm (BD Biosciences), according to the supplier recommendations. For intramembrane staining of CD68, 0.1 μg mouse anti-rat CD68 AF647 (AbD Serotec) were added to this mix and incubated for 25 min at 4°C. After centrifugation, cells were re-suspended in 200 μL working buffer. Cell marker expression was then assessed by flow cytometry with MACS Quant cytometer (Miltenyi Biotec) and data were analyzed using VenturiOne 6.0 software (Applied Cytometry, Dinnington, United Kingdom).

#### Assessment of *in vivo* Fungal Load

Twenty-five milligrams of tissue were re-suspended in 80 μL PBS. The suspension was then grinded with 0.1 mm glass beads (Ozyme) using Cryolys/Precellys 24 (Bertin Instruments) system, for two 25 s cycles at 6,800 rpm. Subsequent DNA extraction steps were performed according to QIAmp DNA Mini Kit (Qiagen) recommendations. *A. fumigatus* 28S ribosomal DNA 28S then quantified by qPCR. Forward primer TCCTCGGTCCAGGCAGG, reverse primer CTCGGAATGTATCACCTCTCGG, and probe (FAM-TGTCTTATAGCCGAGGGTGCAATGCG-BHQ1) were used to perform a Taqman qPCR, with Platinum Quantitative PCR SuperMix-UDG (Thermo Fisher Scientific) on LightCycler 480 (Roche) system. In parallel, a calibration curve was obtained with serial dilutions of DNA (1 × 10^0^ to 1 × 10^7^ fg/μL) from *A. fumigatus* Crf+ strain cultivated *in vitro*. A program with a decontamination of 2′ at 50°C, followed by an initial denaturation of 10′ at 95°C and 50 cycles (15″ at 95°C and 1′ at 60°C) was then used for the amplification.

### Ethics Statement

This study was carried out in accordance with the principles of the Basel Declaration. The rat model of invasive aspergillosis was approved by the General Direction for Research and Innovation, French Ministry of Higher Education and Research through the accreditation number No. 01901.01, and by the Ethics Committee for Animal Experimentation of the Val-de-Loire region through the accreditation number No. C37-261-3.

Collection of human samples was approved by the ethics committee of CHRU of Tours under research project number No. 2016-003. No medical intervention relative to this research protocol was necessary, and the collection of the samples did not affected patient healthcare. Thus, the ethics committee only required informed consent of the patients, with no need of written consent.

### Statistical Analysis

Statistical analyses were performed with XLSTAT 2016.02 (Addinsoft, Paris, France) software. Mann–Whitney test was applied. The α-risk was set to 0.05.

## Results

### Anti-Crf2 MS112-IIB1 Antibody Is Able to Bind to Other Protein Isoforms Coded by *CRF1* Gene

Based on the previous data published by Schütte et al. (2009), we selected a high affinity single chain fragment variable (scFv) antibody (MS112-IIB1, high affinity ∼10^-10^ M) that was obtained using rCrf2 recombinant protein as immunogen.

**FIGURE 1:**
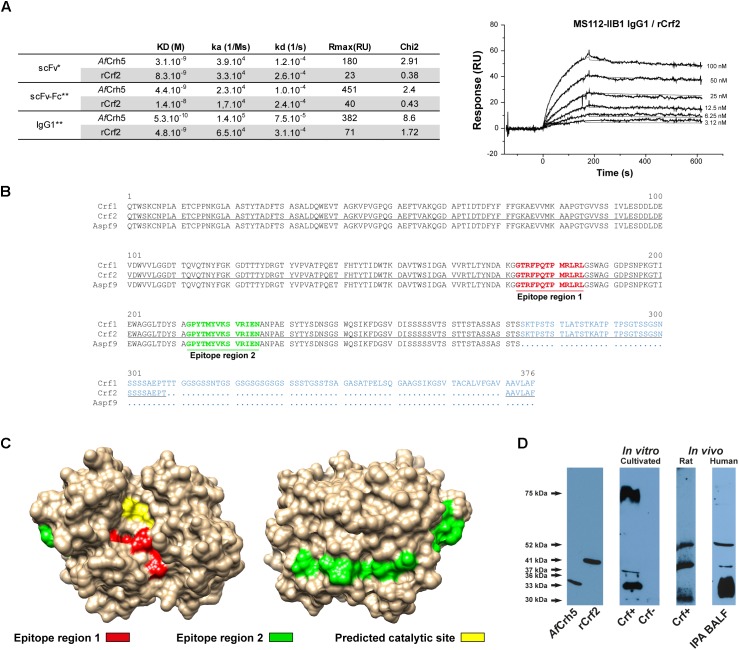
MS112-IIB1 anti-Crf antibody binds to different Crf isoforms. **(A)** Surface plasmon resonance kinetic analysis of MS112-IIB1 scFv, scFv-Fc, and IgG1 binding to immobilized rCrf2 or *Af*Crh5 recombinant proteins. Kinetic and affinity constants are summarized in the table. Sensorgrams were fitted globally using a Langmuir 1:1 model (^∗^) and bivalent model (^∗∗^) for scFv and scFv-Fc/IgG1, respectively. An example of sensorgram is displayed for IgG1 and Crf2 (thin lines are the fitting curves). Rmax: maximum binding level of analyte. Chi2: Chi-square residual value (measure of the average deviation of the experimental data from the fitted curve; lower Chi2 values indicate a better fit). **(B)** Epitope mapping of anti-Crf MS112-IIB1 antibody using a peptide array (15mer oligopeptide overlapping, by 12 amino acid residues). Epitope regions were highlighted in bold red (region 1) or green (region 2) on an alignment of Crf1, Crf2, and Asp f9 protein sequences. Divergent amino acid regions of the three proteins are displayed in blue. Crf2 sequence used for the design of oligopeptides is underlined. **(C)** Localization of epitope regions recognized by MS112-IIB1 on *Af*Crh5 structure. Red: epitope region 1. Green: epitope region 2. Yellow: predicted catalytic site. **(D)** Study of Crf protein isoforms recognized by MS112-IIB1 antibody *in vitro* and *in vivo*, by Western blot. Cell wall proteins, from *A. fumigatus* Crf+ or Crf- (natural mutant) strains cultivated *in vitro* or inoculated in rats, or from human broncho-alveolar lavage fluid of patient with IPA (IPA BALF), were extracted and analyzed by Western blot, using MS112-IIB1 antibody at 2 μg/mL. MS112-IIB1 scFv-Fc primary antibody and anti-human IgG secondary antibody were used for the identification of Crf proteins. Recombinant *Af*Crh5 and Crf2 (rCrf2) proteins were used as control.

Next, the antibody fragment was engineered for therapeutic purpose, adding a Fc (Fragment crystallizable) part to favor recruitment of immunity effectors (Supplementary Figure S1). The affinity of scFv-Fc and IgG1 formats was assessed by surface plasmon resonance (Figure 1A) for both recombinant rCrf2 protein and a protein (*Af*Crh5) containing the common sequence of Crf protein variants (Supplementary Figure S2). ScFv-Fc had a slightly lower affinity for rCrf2 compared to scFv (1.4 × 10^-8^ M vs. 8.3 × 10^-9^ M), while the affinity of IgG1 format was increased (4.8 × 10^-9^ M). Moreover, MS112-IIB1 had a higher affinity for *Af*Crh5 recombinant protein than for rCrf2, whatever the antibody format considered, which suggests that MS112-IIB1 binds to an epitope shared by Crf protein variants. To determine the epitope recognized by MS112-IIB1, we designed 15-mer overlapping oligopeptides covering the entire sequence of Crf2 protein. We found that MS112-IIB1 binds to two peptide regions FPQTPMRLRLGS and GPYTMYVKSVRIENA, which are located in the common sequence of Crf protein variants (Figure 1B). While the first epitope region was located near to the predicted catalytic site of *Af*Crh5, the second was located on the opposite side (Figure 1C). Overall, our results indicate that MS112-IIB1 may be able to recognize all Crf variants. Accordingly, MS112-IIB1 detected, in Western Blot, both recombinant *Af*Crh5 and rCrf2 proteins and several bands against *A. fumigatus* cell wall extracts (Figure 1D). The extended 75 kDa band visible in some experiments (Figure 1D) is probably due to an incomplete digestion of fungal cell wall by beta-glucanase, in which some sugar residues could be still bound to proteins (data not shown). The lower bands couldn’t be related to a specific Crf isoform, due to differences of migration with recombinant proteins and discrepancies with regard to the transcription levels (Figure 1D and Supplementary Figure S2A). Therefore, MS112-IIB1 will be called “anti-Crf antibody” thereafter throughout the text.

### *CRF1* Gene Is Highly Conserved in *A. fumigatus* Strains

To determine whether Crf proteins and the epitopes targeted by anti-Crf antibody were conserved to allow its use in a therapeutic purpose, we sequenced *CRF1* gene in 49 isolates of *A. fumigatus* recovered from routine clinical practice (Table 1). Overall, 45 strains (92%) showed a 100% conserved sequence of the 1303 nucleotides of *CRF1* gene (Supplementary Figure S3). Silent mutations without an effect on the amino acid sequence were found in two strains (4%). Three strains (6%) exhibited missense mutations without affecting epitopes regions recognized by anti-Crf antibody; among them, one showed three changes in amino acid sequence. One strain (2%) exhibited a deletion of two successive nucleotides at the beginning of *CRF1* gene (position 58–59), inducing a frameshift mutation and the emergence of a Stop codon at amino acid 75 in all Crf proteins (referred to as “Crf-” strain). All 49 strains studied (100%) shared the same mutation in nucleotide position 1110, when compared with the published sequence of Af293 strain (NCBI accession number NC_007194), with the replacement of a cytosine (Af293) for a guanine (other strains), inducing a missense mutation (replacement of a threonine by a serine residue) (Supplementary Figure S3). All the mutations did not affect epitope regions recognized by anti-Crf antibody, therefore allowing it to bind to *CRF1* gene products in different pathophysiological conditions, when they are expressed.

**Table 1:** Sequencing of *CRF1* gene on clinical strains of *Aspergillus fumigatus* and assessment of Crf expression by immunofluorescence.

Mutation type	Proportion of strains	Strain name	Detail of mutated strains		IF positive strains
			Nucleotide changes	Amino acid changes	
None (conserved)	45/49 (92%)	Crf+	/	/	45
		IPA 2			
		ABPA 2			
		CF 2			
Silent mutation	2/49 (4%)^∗^		Mutation in position 190 (C→T)	Silent mutation in codon 43	2^∗^
			Mutation in position 1138 (C→T)	Silent mutation in codon 341	
Missense mutation	3/49 (6%)^∗^	ABPA 1	Mutation in position 629 (G→T)	Amino acid change in codon 172 (Val→Phe)	3^∗^
			Mutation in position 927 (T→C)	Amino acid change in codon 271 (Ile→Thr)	
		CF 1	Mutation in position 927 (T→C)	Amino acid change in codon 271 (Ile→Thr)	
			Mutation in position 1124 (A→T)	Amino acid change in codon 337 (Thr→Ser)	
			Mutation in position 1126 (C→T)		
Frameshift mutation	1/49 (2%)	Crf-	Deletions in positions 58-59	Open reading frame shift	0
				Change of all amino acids from codon 20	
				Stop codon emergence in codon 75	


*Forty-nine isolates of *A. fumigatus* were collected at the CHRU of Tours (France), from respiratory samples of patients without development of aspergillary disease (*n* = 38), or affected by ABPA (*n* = 4), invasive pulmonary aspergillosis (*n* = 3), aspergilloma (*n* = 1), *Aspergillus* bronchitis (*n* = 1), or from environmental contamination (*n* = 2). Groups indicated with (^∗^) contain a common strain that cumulates silent and missense mutations. IF, Immunofluorescence.*

### Crf Protein Is Expressed *in vitro* and *in vivo*, in Different Pathophysiological Conditions

**FIGURE 2:**
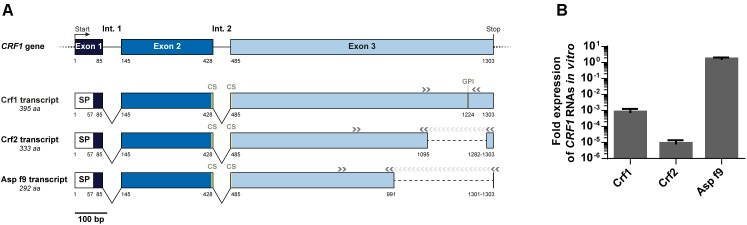
Expression of *CRF1* alternative transcripts in *A. fumigatus*. **(A**) Schematic representation of *CRF1* gene and the three mRNA transcripts, Crf1, Crf2, and Asp f9 with the corresponding primers used for amplification. Nucleotide positions of the beginning/end of each exon (boxes) and intron (Int., in full lines) are mentioned under the gene and the transcripts. Non-coding regions are identified in dotted lines. SP: position of predicted signal peptide. CS: position of predicted catalytic site. GPI: position of predicted GPI anchor. Position of primers used for the amplification of the different transcripts is indicated by gray arrows (>). **(B)** *In vitro* expression of Crf1, Crf2 and Asp f9 RNA transcripts in RT-qPCR. Transcript expression was analyzed in Crf positive strain by RT-qPCR and normalized toward gpdA (glutaraldehyde 3-phosphate dehydrogenase) and TUB1 (beta-tubulin) housekeeping genes, set to 1. Results (*n* = 5) are expressed in mean ± SD.

**FIGURE 3:**
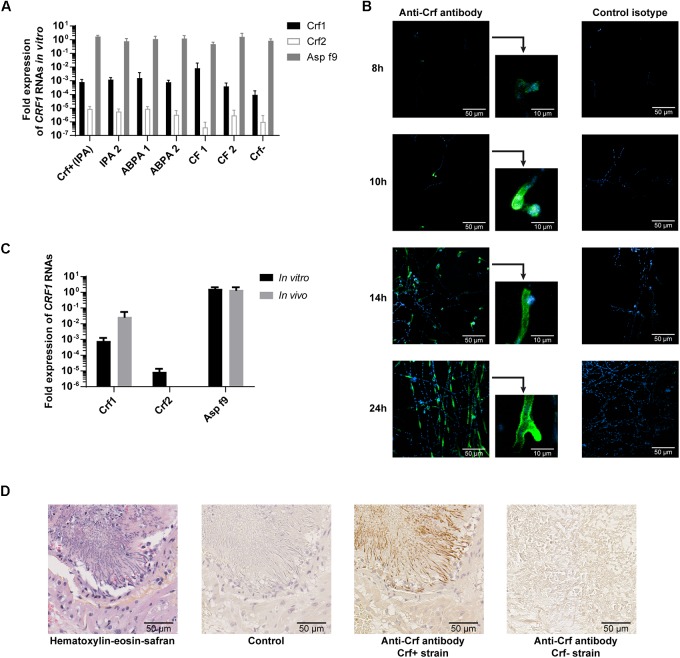
Expression of Crf transcripts and isoforms in clinical strains, *in vitro* and *in vivo*. **(A)** *CRF1* RNA transcript expression in different clinical strains, by RT-qPCR. Results were normalized toward *gpdA* and *TUB1* housekeeping genes, set to 1. Results are expressed in mean ± SD. IPA, strains from patients with invasive pulmonary aspergillosis (including Crf+); ABPA, strains from patients with allergic bronchopulmonary aspergillosis; CF, strains from patients with colonization (cystic fibrosis); Crf-, clinical strain with natural frameshift mutation, with no expression of Crf proteins. **(B)** Expression kinetic and localization of Crf proteins (in green) *in vitro* by immunofluorescence in confocal microscopy. MS112-IIB1 scFv-Fc anti-Crf antibody (or isotype) at 2 μg/mL were used. Cell nuclei were stained in Hoechst (in blue). Scale bar: 50 μm or 10 μm. Original magnification: ×400. **(C)** Differential expression of *CRF1* RNA transcript *in vivo* and *in vitro*, by RT-qPCR. RNAs were extracted Crf+ strain cultured *in vitro* or from lungs of rats infected by Crf+ strain. Results (*n* = 5) are expressed in mean ± SD. **(D)** Expression of Crf proteins (in brown) *in vivo*, in rat lungs tissues, by immunohistochemistry. Slides of rat lungs either infected by Crf+ or Crf- (natural mutant) strain were studied with MS112-IIB1 scFv-Fc antibody at 2 μg/mL or control. One slide was treated in HES coloration. Scale bar: 50 μm. Magnification: ×400.

To study the expression of *CRF1* gene, we designed primers to specifically detect each *CRF1* alternative transcripts (encoding Crf1, Crf2, and Asp f9) and assessed their quantitative expression by RT-qPCR (Figure 2A). As shown for the reference *A. fumigatus* strain Crf+ (Figure 2B), Asp f9 was the most expressed transcript; Crf1 RNA expression was lower by about 10^3^-fold than for Asp f9 and Crf2 was the less expressed transcript with a difference of 10^5^-fold compared with Asp f9. The RNA expression profile of Crf1, Crf2 and Asp f9 transcripts isolated from IPA patients (*n* = 2), ABPA patients (*n* = 2) or CF colonized patients (*n* = 2) was similar to Crf+ strain (Figure 3A).

Next, we studied *CRF1* gene expression at the protein level. The strains (*n* = 6) exhibited a similar profile than Crf+ strain in Western blot (data not shown), and we detected Crf proteins in 98% of *A. fumigatus* clinical isolates (*n* = 48) – except for Crf- strain – as shown by immunofluorescence (Table 1). Kinetics indicated that Crf proteins began to be detectable after 8 h incubation with growing conidia (Figure 3B). Expression was initially localized in budding, and then in fungal cell wall, and in *septa* when they began to develop at around 14 h growth. Location of Crf proteins was similar in all *A. fumigatus* clinical isolates, except Crf- strain (data not shown).

As *in vitro* growth is not representative of *in vivo* conditions (Hartmann et al., 2011), we then assessed the expression of Crf+ strain *CRF1* RNA transcripts in a rat model of aspergillosis. *In vivo*, we highlighted a difference of expression of Crf1 and Crf2 transcripts *vs*. *in vitro* (Figure 3C). While the expression of the former was increased by 34-fold *in vivo*, the latter was not detected *in vivo*. Expression of Asp f9 was similar in both conditions. We confirmed the expression of Crf proteins in the rat model by Western blot, which also showed a different profile than *in vitro* (Figure 1D). Mass differences may be attributed to posttranslational modifications (i.e., glycosylations), as previously described (Leach and Brown, 2012) and predicted for Crf proteins *in silico* (not shown). We then studied the *in vivo* expression of Crf proteins in the lungs of infected rats by immunohistochemistry. Crf+ strain exhibited a specific signal located in areas of fungal growth, on the periphery of infectious foci, where hyphae invade surrounding tissues (Figure 3D). Moreover, the expression of Crf proteins in lung tissues from a patient with IPA was also observed by immunohistochemistry (Supplementary Figure S4). Finally, the study of the expression of Crf proteins in broncho-alveolar lavage fluid collected from a patient suffering of IPA exhibited a similar profile to the profile observed in the rat model of IPA (Figure 1D).

### Deletion of *CRF1* Gene Reduces *A. fumigatus* Growth

**FIGURE 4:**
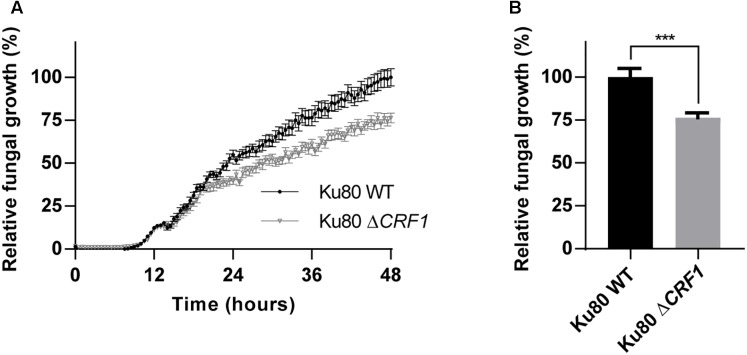
Growth of *CRF1* engineered mutant strain *in vitro*. **(A)** Assessment of the consequences of the absence of *CRF1* gene (coding Crf1, Crf2, and Asp f9 proteins) on fungal growth. A growth kinetic was realized on Ku80 and Ku80 Δ*CRF1* strains with a reading of 530 nm absorbance every 30 min, during 48 h. **(B)** Growth differences at 48 h. Results (*n* = 15) are expressed in mean ± SEM; Mann–Whitney statistical test was used. ^∗∗∗^*p* < 0.001.

To characterize the role of Crf proteins on the fungal growth we used a knock-out mutant deleted for *CRF1* gene, named Δ*CRF1* (Supplementary Figure S5). A growth study revealed a slower growth over time for mutant compared to wild type strain, with a difference visible from 15 h incubation (Figure 4A). At 48 h, mutant growth was significantly altered (*p* < 0.001) with a decrease by 23.6% (Figure 4B).

### Anti-Crf Antibody Neutralizes Enzymatic Activity of Crf and Reduces Fungal Growth *in vitro*

**FIGURE 5:**
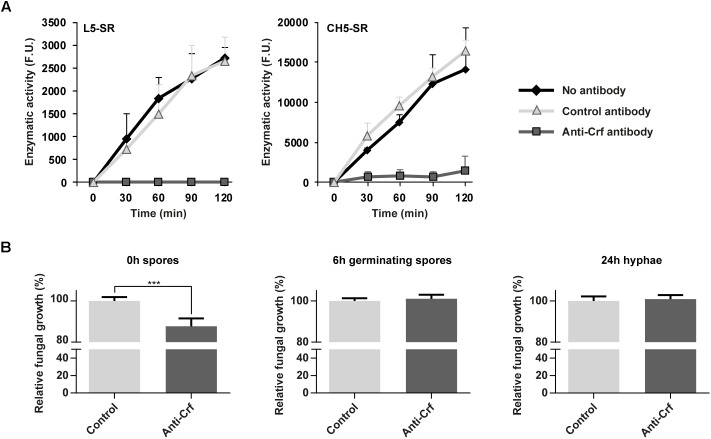
Neutralization of Crf proteins *in vitro*, by enzymatic assay and fungal growth inhibition assay. **(A)** Neutralization of recombinant Crf enzymatic activity by anti-Crf IgG1 antibody. The rates of transglycosylation catalyzed by *Af*Crh5 recombinant protein were determined using 18 μM sulforhodamine-labeled oligosaccharides (laminaripentaose L5-SR or chitopentaose CH5-SR) as acceptor and carboxymethyl-chitin (0.1%) as a donor in 50 mM citrate buffer pH 5.8. *Af*Crh5 was pre-incubated in PBS (No antibody), IgG1 trastuzumab anti-HER2 antibody (Control antibody), or IgG1 MS112-IIB1 antibody (Anti-Crf antibody) at 2:1 (antibody:*Af*Crh5) molar ratio. Data represent the media and standard deviation of at least three independent experiments. F. U., arbitrary fluorescence units. **(B)** Neutralizing effects of anti-Crf IgG1 antibody on fungal growth. Anti-Crf IgG1 antibody or control antibody were added on Crf+ strain fungal cultures of 0 h, 6 h or 24 h, at a final concentration of 0.5 μg/mL. Absorbance at 530 nm was read after a 48 h incubation. Results (*n* = 16) are expressed in mean ± SEM; Mann–Whitney statistical test was used. ^∗∗∗^*p* < 0.001.

Given the involvement of *CRF1* gene in *A. fumigatus* growth, we investigated whether anti-Crf IgG1 antibody may neutralize *Af*Crh5 protein enzymatic activity and decrease fungal growth *in vitro*. Using a fluorescence *in vitro* enzymatic assay (Mazan et al., 2013), the transglycosylase activity of *Af*Crh5 protein was measured using sulforhodamine (SR)-labeled oligosaccharides derived from β-1,3 glucan (L5-SR) and chitin (CH5-SR) as acceptors and carboxymethyl-chitin (CM-chitin) as donor. *Af*Crh5 showed transglycosylation activity with both substrates (Figure 5A). Moreover, when compared to an unrelated control antibody (trastuzumab), the anti Crf antibody exhibited a complete inhibition of the transglycosylase activity with both acceptors (Figure 5A). In addition, antibody recognized epitopes have been shown to be very close to the predicted catalytic site of Crf protein (Figure 1C).

Anti-Crf antibody was then tested *in vitro* on *A. fumigatus* cultures (Figure 5B) of spores (0 h growth), germinating spores (6 h growth) and hyphae (24 h growth). Compared to irrelevant antibody (control isotype trastuzumab), after a 48 h-long culture, anti-Crf IgG1 antibody elicited a decreased growth of 12.3% (*p* < 0.001) when added onto 0 h spores, but with no visible structural change to microscopic observation. Growth difference was observable from 15 h incubation (Supplementary Figure S6A). No neutralization effect was observed when anti-Crf was added on 6 h germinating spores or 24 h hyphae, suggesting that Crf inhibition is not sufficient to block *A. fumigatus* growth. Studies with scFv-Fc antibody format gave similar results (Supplementary Figure S6B).

### Treatment With Anti-Crf Antibody Stimulates the Recruitment of Immune Cells *in vivo* but Does Not Inhibit Fungus Growth

Because anti-Crf antibody (IgG1 format) contained a Fc domain that may favor the recruitment of immune effectors and help to eliminate the fungus, we then examined the effects of anti-Crf antibody in a rat model of aspergillosis.

**FIGURE 6:**
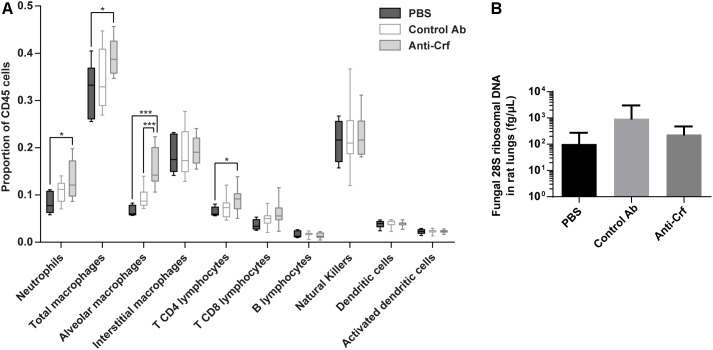
Anti-Crf IgG1 antibody effects in a rat model of IPA. Neutropenic rats were infected by 10^5^ spores of Crf+ strain, and challenged extemporaneously either with intra-tracheal aerosolization of PBS (*n* = 6), control antibody (Control Ab, *n* = 12) or anti-Crf IgG1 antibody (*n* = 12). Antibody were administered twice (4 mg/kg), with the spores and 32 h post-infection. Rats were sacrificed 72 h after the infection. **(A)** Evaluation of IgG1 effects on the recruitment of immune cells on infected rat lungs. Immune cells were extracted from lungs and processed in flow cytometry. Neutrophils (CD11b^hi^, Ly6G^hi^), total macrophages (CD11b^hi^, Ly6G^low^), alveolar macrophages (CD11b^hi^, Ly6G^low^, CD172^hi^, CD68^hi^), interstitial macrophages (CD11b^hi^, Ly6G^low^, CD172^low^, CD68^hi^), T CD4 lymphocytes (CD4^+^, TCR^+^), T CD8 lymphocytes (CD8a^+^, TCR^+^), B lymphocytes (CD45R^+^, IgM^+^), natural killers (KLRB1^+^), dendritic cells (CD11c^hi^, MHC-II^hi^) and activated dendritic cells (CD11c^hi^, MHC-II^hi^, CD86^hi^) populations were studied. Results are expressed in median ± interquartile (box) with min/max (bars); Mann–Whitney statistical test was used. ^∗^*p* < 0.05, ^∗∗∗^*p* < 0.001. **(B)** Evaluation of IgG1 effects on the lung fungal load of a rat model of IPA. Fungal DNA was extracted from lungs and *A. fumigatus* load was assessed by assaying 28S ribosomal DNA by qPCR. Results are expressed in mean ± SD; Mann–Whitney statistical test was used.

First, we investigated immune cell recruitment in the lungs by flow cytometry (Figure 6A and Supplementary Figure S7A). Animals treated with anti-Crf IgG1 antibody showed a significant increase in the recruitment of neutrophils (increase by 59% compared to PBS, *p* < 0.05), total macrophages (increase by 23% compared to PBS, *p* < 0.05), alveolar macrophages (increase by 134% compared to PBS, 63% compared to irrelevant antibody, *p* < 0.001) and T CD4 lymphocytes (increase by 42% compared to PBS, *p* < 0.05) compared to the control groups (PBS and irrelevant control antibody trastuzumab). The other cell populations remained unchanged.

However, at the time of sacrifice, no difference was observed in the lung fungal load between PBS group (1.0 × 10^2^ fg/μL), irrelevant antibody group (9.5 × 10^2^ fg/μL) and anti-Crf IgG1 antibody group (2.3 × 10^2^ fg/μL) (Figure 6B). Testing of scFv-Fc antibody format gave similar results (Supplementary Figure S7B). There was no statistical difference regarding overall survival between control groups (aerosolized intra-tracheally with either PBS or irrelevant control antibody) and treated group (treated with anti-Crf scFv-Fc antibody). Survival time in all groups did not exceed 120 h post-infection.

## Discussion

*Aspergillus fumigatus* is an environmental mold fungus which is responsible for invasive aspergillosis, a life-threatening infection that is usually encountered in immunocompromised hosts. Current treatments against aspergillosis suffer from either limited efficacy or deleterious side effects, or both, which therefore restricts their use. While several antibodies have been raised against this fungus for a diagnostic purpose, only a few have been developed for therapeutic applications (Chaturvedi et al., 2005, 2009; Schubert et al., 2018). Therapeutic antibodies are emerging as a new class of anti-infectious agents and may be suited for treatment against *A. fumigatus* (Kuhn et al., 2016; Sécher et al., 2018; Wagner and Maynard, 2018). Usually, therapeutic antibodies target highly immunogenic antigens that are expressed on the cell surface. Herein, we assessed the relevance of Crf CWPs, which have been known for years to be allergens and have been quite successfully used in vaccination assays (even considering the limitation of immunocompromised context), as molecular targets for antibodies to treat aspergillosis (Ramadan et al., 2005; Medici and Poeta, 2015). According to our results discussed below, Crf proteins and anti-Crf antibodies display several benefits and fulfill several requirements for the development of anti-infectious therapeutic strategy.

Firstly, our findings indicate that Crf proteins are suitable molecular targets for antibody-based therapy because of the high conservation of *CRF1* gene within *A. fumigatus* specie. If less than 10% of the studied clinical strains exhibited mutations, most of them had a limited impact on protein expression; only one isolate showed a mutation resulting in an early codon stop in *CRF1* gene (Crf-mutation). Secondly, Crf proteins are highly and constantly expressed. The *in vitro* study by RT-qPCR and Western blotting successfully highlighted the homogenous production of Crf transcripts, whatever the clinical origin of the tested strains. Whereas Asp f9 was strongly expressed, Crf2 expression was found to be very low, therefore it can explain why this transcript has not been identified so far in many previous works. In the rat model of IPA, Crf1 and Asp f9 were also found to be expressed at a high level by RT-qPCR. Unfortunately, Crf1, Crf2 and Asp f9 isoforms could not be linked to the bands observed in the Western blot, because of inconsistencies in molecular weights and previously described transcription levels; use of mass spectrometry should allow their identification. Crf proteins were expressed on germinating spores and hyphae of *A. fumigatus*, the latter being the representative stage of clinical aspergillosis. As for the *in vitro* experimentations, *in vivo* localization of Crf proteins was found to be heterogeneous but maximal in the regions of fungal growth, at the surface of buddings and *septa* areas (Arroyo et al., 2007; Schütte et al., 2009). Crf proteins were mostly expressed in the periphery of infectious foci, which is consistent with the fact that the fungus is more metabolically active at these sites (Willger et al., 2009). Infectious foci usually grow from the center to the outer region, theoretically thus enabling easy access to therapeutic antibodies, independently of the route of administration. At last, Crf proteins were proven to play a structural role in *A. fumigatus* growth. Indeed, engineered knock-out mutant for *CRF1* gene (Δ*CRF1*) exhibited a significant but modest decrease in fungal growth, visible after a 15 h-culture. This finding may be due to a loss of *A. fumigatus* cell wall integrity, knowing the suspected action of Crf in the linkage of chitin to β(1-3)glucans. The relative limited impact of Crf proteins in *A. fumigatus* growth may be attributed to a compensation by ortholog proteins with the same function (Crh1, Crh2, Crh3, and Crh4) or overproduction of other cell wall components (Arroyo et al., 2016). All the aforementioned reasons led us to consider Crf proteins as relevant antigens for anti-Crf therapeutic antibodies. However, engineering of anti-Crf fragment antibodies (with only antigen binding site) in full-length antibodies would be required to optimize their anti-fungal activity, mediating effector functions through the Fc domain, as previously demonstrated for other therapeutic antibodies (Maloney et al., 2002; Mayr et al., 2017).

In addition, we demonstrated *in silico*, *in vitro*, and *in vivo* that anti-Crf antibodies display several advantages and may be used in *A. fumigatus* infection. First, anti-Crf antibodies are highly specific. As previously shown by Schütte et al. (2009) and based on sequence alignments of Crf proteins (data not shown), it is most likely that anti-Crf antibodies would not exhibit cross reactivity toward other *Aspergillus* species or against yeasts like *Candida albicans* (Schütte et al., 2009). Moreover, anti-Crf antibodies did not show cross reactivity against known Crf orthologs Crh1, Crh2, Crh3, and Crh4, as demonstrated by the absence of unspecific bands in the Western blot for the engineered mutant of Crf. Finally, anti-Crf-antibody did not show off-target binding, but they showed a high affinity, which increased after the format modification from scFv into IgG1. Epitope mapping indicated that anti-Crf antibodies recognize the distinct Crf isoforms encoded by *CRF1* gene, since epitope regions were found to be located in the common regions for Crf1, Crf2 and Asp f9; this was supported by Western blotting and surface plasmon resonance. We also demonstrated the neutralizing property of anti-Crf antibodies, as demonstrated by their capacity to inhibit the enzymatic activity of a recombinant *Af*Crh5 protein. This is consistent with the location of the first epitope region (epitope 1), which was very close to the predicted catalytic site of epitopes in the *Af*Crh5 structure. To date, there is insufficient information to determine whether the antibodies neutralize Crf enzymatic activity through steric hindrance or through induction of a conformational modification of the protein structure, which is a possibility for the second epitope region (epitope 2). In light of these initial results, anti-Crf antibodies were then tested *in vitro* on fungal cultures. Unfortunately, they only enabled partial inhibition of fungal growth when added to a spore suspension, but had no action against germinating spores or hyphae. Despite the fact that Crf proteins were sometimes found secreted (Wartenberg et al., 2011), it is unlikely that the limited effect of anti-Crf antibodies might be due to sequestration by soluble Crf since increasing the amount of antibody did not result in a higher inhibitory effect, *in vitro* (not shown). Considering the expression kinetics of Crf, a very early therapeutic action is probably required in order to act efficiently on the fungus growth. Administration of the antibodies at 0 h could allow early neutralization of Crf proteins, as soon as they are expressed at the beginning of the germination. Considering the limited inhibition of anti-Crf antibody *in vitro* – associated to its Fab part only –, we tested full-length anti-Crf antibody activity in a rat model of IPA. We revealed the ability of their Fc part to recruit immune cells *in vivo*, with the significant demonstration of a major and increased recruitment of neutrophils, total macrophages and alveolar macrophages. As neutrophils and macrophages are the first defense barrier against *A. fumigatus* (Dagenais and Keller, 2009), their recruitment, could take place *via* ADCC (antibody dependent cell-mediated cytotoxicity) or ADCP (antibody dependent cell-mediated phagocytosis) mechanisms, thus suggesting that anti-Crf antibodies are able to stimulate the most important components of the natural immune response during aspergillosis. Even if the adaptive response is quite limited in IPA, increased stimulation of the CD4^+^ T-lymphocytes population at time of the sacrifice (72 h) is consistent with the usual kinetics of recruitment of such cells during natural recovery from infection (Bozza et al., 2002; Rivera et al., 2005). Local attraction of CD4^+^ T-cells in infected lungs could have led to the reinforcement of neutrophils and macrophage recruitment with the production of cytokines and chemokines. Surprisingly, no significant differences were observed in the recruitment of dendritic cells (including activated), even though these cells (among other subsets) are known to be at the origin of CD4^+^ T-cells activation in lymphoid nodes through antigen presentation process (Rivera and Pamer, 2009), inducing either a Th1 (protective), Th2 (deleterious) or Th17 (still controversial) differentiation profile (Dewi et al., 2017). Noteworthy, the therapeutic effect of anti-Crf antibodies was insufficient to decrease the fungal burden in our rat model of IPA and to improve significantly the overall survival.

Although Crf proteins and anti-Crf antibodies meet most of the criteria for an ideal antigen target and ideal therapeutic antibodies, antifungal impact was not sufficient to efficiently inhibit fungal growth *in vivo*. Such a strategy is, however, relevant, as shown by previous studies where *in vivo* testing of IgG or IgM against CWPs demonstrated a decrease in fungal burden, which was consistent with a therapeutic effect (Chaturvedi et al., 2005, 2009). The limited effect of anti-Crf antibodies may be due to several parameters: first, the rat model of IPA we used to assess the effects of anti-Crf antibodies *in vivo* (Chandenier et al., 2009). Given IPA epidemiology, we selected a neutropenic model rather than a steroid treated model. Neutropenia context represents more than 50% of clinical cases, including patients with acute leukemia and chronic lymphoproliferative disorders for which the mortality rate is the higher (Lortholary et al., 2013). However, in spite of its high reproducibility, the animal model may be quite questionable because it usually leads rapidly to death within 2–4 days after the infectious challenge. One can suspect that this time-lapse was too short to thoroughly assess the therapeutic effects of anti-Crf antibodies (Desoubeaux and Cray, 2017). Another hypothesis has been raised concerning the antibody engineering: even if human IgG1 has been described to interact with rat Fc gamma receptor of rat macrophages (Boltz-Nitulescu et al., 1981) – which we confirmed in this study by the ability of our anti-Crf antibodies to recruit several populations of immune cells –, this interaction may be insufficient to prevent the development of the infection. In such a case, further engineering of the Fc part (modulation of fucosylation, galactosylation…) would allow an enhanced interaction (Shields et al., 2002; Thomann et al., 2016). In contrast, no one can argue that the antibody doses used in this study were too low: whereas Chaturvedi et al. (2005) used a mouse model treated with the rat equivalent of 2.5 mg/kg (Nair and Jacob, 2016) in intravenous single dose to demonstrate the anti-*Aspergillus* activity of their Mab A9 antibody, we used two repeated doses of 4 mg/kg, administrated *in situ*, directly into the airways (Chaturvedi et al., 2005). Local delivery of antibodies through the airways was probably the optimal route to achieve a high concentration of the macromolecule within the lungs, where the fungus is primarily located (Guilleminault et al., 2014; Respaud et al., 2015).

Finally, we can hypothesize that Crf proteins themselves can be the cause of the moderate antifungal activity of the anti-Crf antibodies. The lack of Crf proteins did not result in a totally non-viable phenotype, and we found only a slight – but significant – decrease of fungal growth with the mutant deleted for the *CRF1* gene. Surprisingly, previous studies on *CRF1* mutants did not reveal changes in fungal growth, possibly due to different experimental conditions [Chabane S, Reichard U, unpublished; Fang W, Van Aalten DM, unpublished (Fang et al., 2015)]. These results were also confirmed by the *in vitro* targeting of Crf proteins by our antibodies, which only exhibited a limited growth decrease. During our investigations, we also discovered a natural mutant for the expression of Crf proteins (Crf-) in a clinical strain which did not display obvious phenotypic changes in fungal morphology and growth. Thus, a compensation of Crf activity by an overexpression of other orthologous transglycosylases like Crh1, Crh2, Crh3, and Crh4 is probable, allowing the fungus to keep growing (Arroyo et al., 2016). Thus, combination of antibodies targeting different CWPs of *A. fumigatus* may be relevant to avoid compensatory mechanisms.

Overall, this study demonstrated that Crf transglycosylases are a theoretically relevant target for therapeutic antibodies, because they are ubiquitously expressed by *Aspergillus fumigatus* molds. Use of anti-Crf antibodies, initially developed for a diagnosis purpose against Crf2 protein, did not only exhibit recognition of several Crf isoforms, but also a neutralizing activity against all these enzymes. However, their antifungal effects on fungal growth were moderate, limiting their use for a therapeutic purpose if considered alone. Combination of antibodies with anti-infectious agents may be an attractive strategy as it may lead to a synergistic effect (Al-Hamad et al., 2011; Song et al., 2012), but the interest of associating antibody and antifungal drugs remains to be clearly demonstrated (Richie et al., 2012; Bugli et al., 2013). Cocktail of antibodies targeting several proteins involved in the cell wall construction (as β(1-3)glucans or chitin synthesis enzymes) may avoid escape mechanisms and furnish a less toxic combination therapy to classic chemical molecules in the treatment of IPA. Overall our findings offer future perspective for the design of new anti-*Aspergillus* therapeutics.

## Author Contributions

DC, MH, JA, JC, NH-V, and GD designed the experiments. DC, MS, AS, AC, CP, GM, MP, PM, NH-V, and GD performed the experiments. DC, JC, NH-V, and GD wrote the manuscript.

## Conflict of Interest Statement

NH-V is a founder and a business associate in a CRO called Cynbiose Respiratory, which has no link with the project mentioned in this article. The remaining authors declare that the research was conducted in the absence of any commercial or financial relationships that could be construed as a potential conflict of interest.
