# Supplementary material for: Targeting Aspergillus fumigatus Crf Transglycosylases With Neutralizing Antibody Is Relevant but Not Sufficient to Erase Fungal Burden in a Neutropenic Rat Model
Source: Front Microbiol. 2019 Mar 26;10:600. doi: 10.3389/fmicb.2019.00600 (PMC6443627; doi:10.3389/fmicb.2019.00600)
Supplement: Supplementary file 1 [file Data_Sheet_1.PDF]

Supplementary Material

Targeting *Aspergillus fumigatus* Crf transglycosylases with neutralizing antibody is relevant but not sufficient to erase fungal burden in a neutropenic rat model

David Chauvin, Michael Hust, Mark Schütte, Adélaïde Chesnay, Christelle Parent, Gustavo Marçal Schmidt Garcia Moreira, Javier Arroyo, Ana Belén Sanz, Martine Pugnière, Pierre Martineau, Jacques Chandenier, Nathalie Heuzé-Vourc'h, Guillaume Desoubeaux*

*** Correspondence:** Guillaume Desoubeaux : guillaume.desoubeaux@univ-tours.fr

Supplementary figure count: 7

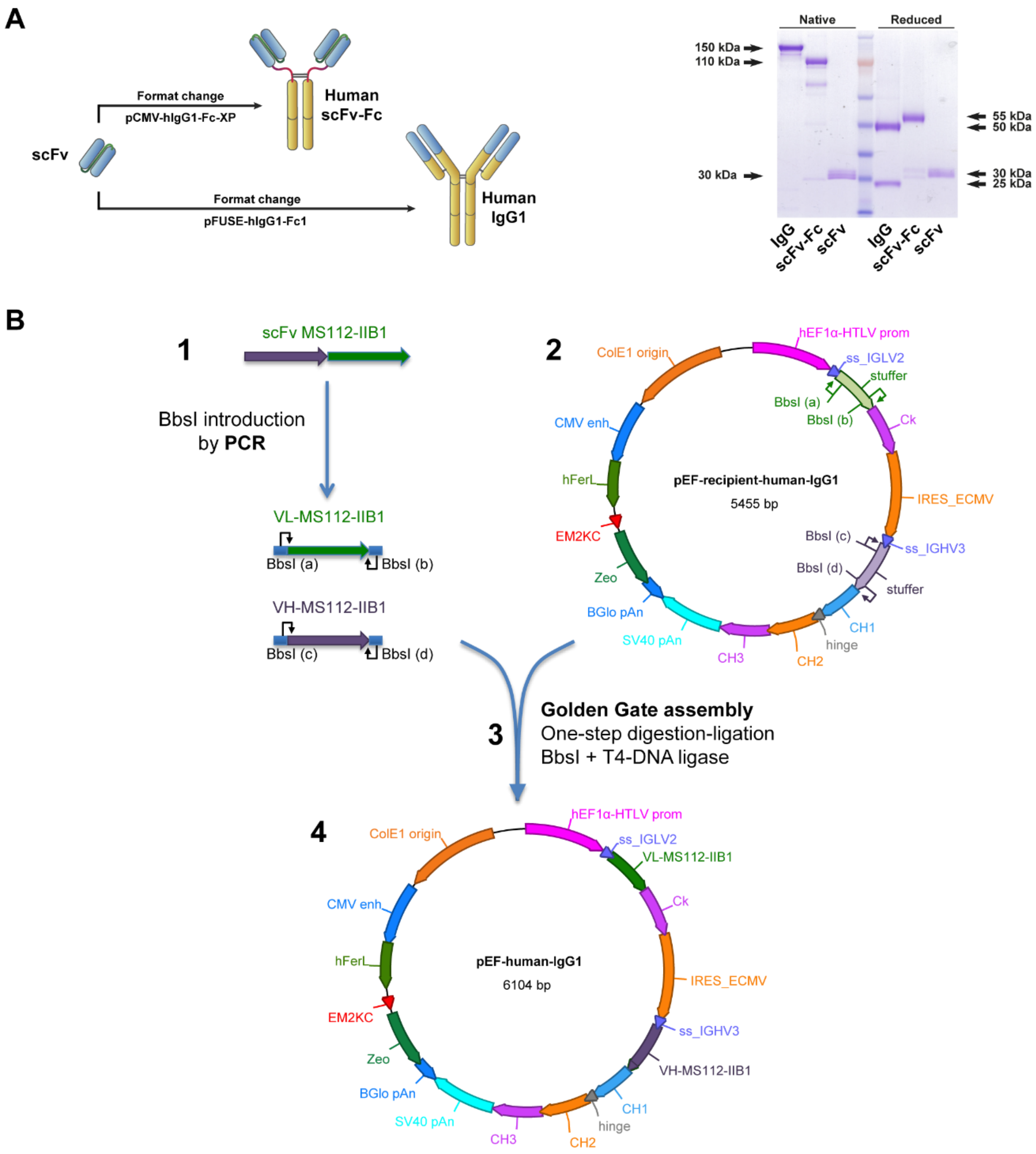

Supplementary Figure S1. Format modification of anti-Crf MS112-IIB1 antibody. (A) Diagram of the format modification of MS112-IIB1 antibody from scFv to human scFv-Fc and human IgG1 (left), and verification of antibody structure in Coomassie blue by SDS-PAGE in native and denaturing/reducing conditions (right). Three micrograms of each antibody were heated and reduced (Reduced) or not heated and not reduced (Native), and dropped on a 4-12% polyacrylamide gel, before a staining in Coomassie blue. **(B)** Schematic view of the cloning steps of MS112-IIB1 IgG1 expression plasmid. The expression plasmid is derived from pFUSE-hIgG1-Fc1 vector (Invivogen, Toulouse, France; https://www.invivogen.com/pfuse-higg1-fc). Expression of the two chains is driven by a composite promoter comprising the Elongation Factor-1α (EF-1α) core promoter and the R segment, and part of the U5 sequence (R-U5') of the Human T-Cell Leukemia Virus (HTLV) Type 1 Long Terminal Repeat in front of the light chain. Initiation of translation of the heavy chain uses the

Encephalomyocarditis virus internal ribosome entry site (IRES_ECMV). Such a bicistronic arrangement for full length IgG expression has been previously described in (Li et al., 2007). 1: First, VH and VL genes were amplified from the scFv gene using primers introducing BbsI sites at each extremities. BbsI enzyme is a type IIS restriction enzyme that recognizes asymmetric DNA sequences and cleave outside of its recognition sequence, leaving a 4-bases 3’ overhang. The four BbsI sites were chosen in order to generate four different non-palindromic overhangs. 2: The same four overhangs (noted a, b, c and d) were generated by BbsI digestion of the recipient plasmid. 3: Simultaneous digestion and ligation of the recipient plasmid and the PCR fragment which can thus be assembled in a single orientation, resulting in the final expression plasmid: 4, as described in (Engler et al., 2008).

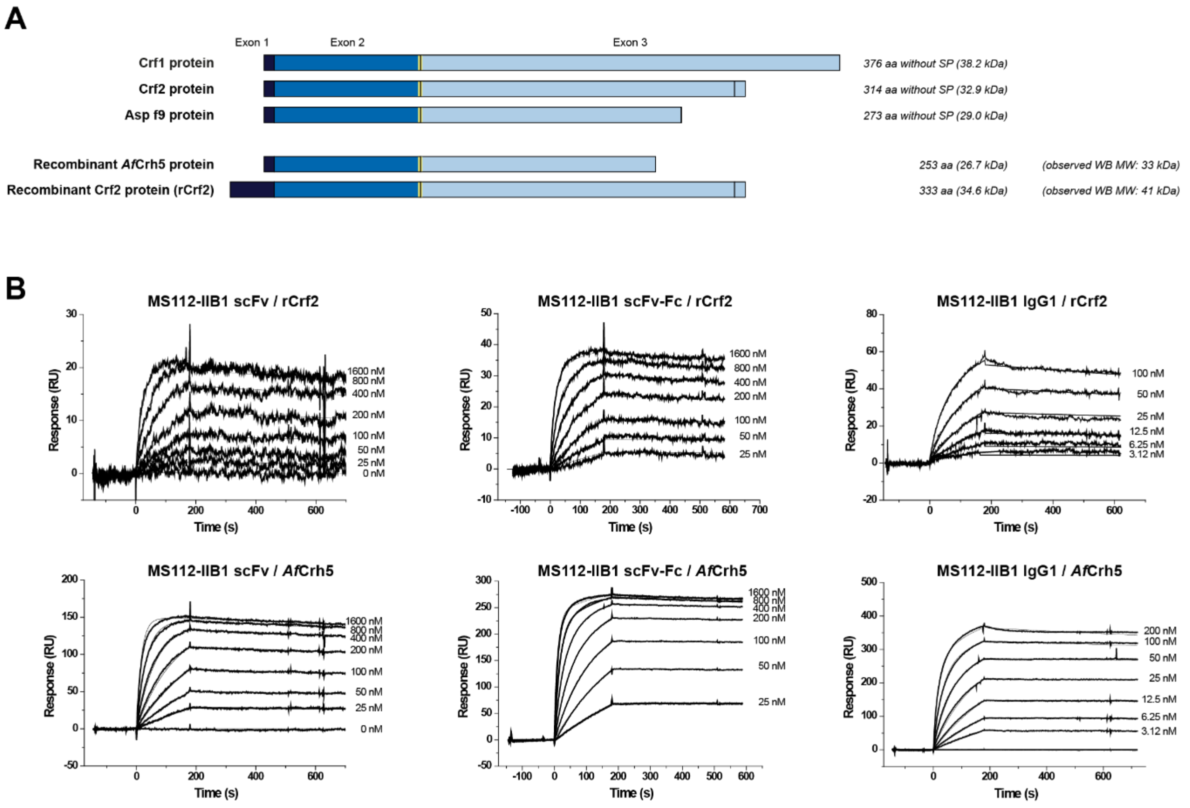

Supplementary Figure S2. Assessment of anti-Crf MS112-IIB1 antibody affinity. (A) Schematic representation of Crf1, Crf2 and Asp f9 proteins and comparison with recombinant proteins *Af*Crh5 and rCrf2. Amino acid length (aa) and corresponding predicted molecular weights (MW) are displayed at the right of each protein. SP: Signal Peptide. WB: Western Blot. Predicted catalytic site is displayed in yellow. **(B)** Surface Plasmon Resonance kinetic analysis of MS112-IIB1 scFv, scFv-Fc and IgG1 binding to immobilized rCrf2 or *Af*Crh5 recombinant proteins. Sensorgrams were fitted globally using a Langmuir 1:1 model (scFv) and bivalent model (scFv-Fc/IgG1). Thin lines are the fitting curves.

```
   1 ATGTATTTCA AGTACACAGC AGCAGCCCTA GCTGCGGTGC TCCCTCTTTG
  51 CTCTGCACAG ACTTGGTCAA AGTGCAATCC CCTTGAGAGT GAGTGTTTTC
 101 ATACCGACAT ATGATATACA TCAGCTTATC TAACGATTGT TTTGCAGAGA
 151 CCTGCCCGCC CAACAAGGGT CTTGCTGCAT CCACTTACAC CGCCGACTTC
 201 ACCTCAGCTT CAGCTTTGGA TCAATGGGAA GTCACTGCAG GCAAAGTTCC
 251 CGTTGGCCCA CAGGGCGCCG AGTTCACTGT CGCTAAGCAA GGCGACGCAC
 301 CTACCATTGA CACCGACTTC TACTTCTTCT TCGGAAAGGC CGAAGTGGTG
 351 ATGAAGGCCG CTCCTGGCAC AGGTGTTGTT AGCAGCATCG TCCTGGAGTC
 401 GGATGATCTG GATGAGGTTG ACTGGGTAAG CCTGCTTGTC TATCATGTGT
 451 TCGTCTTGAG CCGGACTTAA CGAAAGCGCA GGAAGTATTG GGCGGTGACA
 501 CCACTCAGGT TCAGACAAAC TACTTTGGCA AAGGAGACAC CACCACATAT
 551 GACCGAGGCA CTTACGTGCC CGTTGCCACT CCTCAGGAGA CTTTCCACAC
 601 CTACACCATC GACTGGACCA AGGATGCCGT TACCTGGTCT ATTGACGGTG
 651 CGGTCGTGCG TACGCTCACG TACAACGATG CCAAGGGTGG CACTCGCTTC
 701 CCTCAGACTC CTATGCGCCT GAGACTTGGC AGCTGGGCCG GCGGCGACCC
 751 CAGCAACCCC AAGGGCACCA TCGAGTGGGC CGGTGGCTTG ACCGACTACA
 801 GCGCGGGACC GTACACCATG TACGTCAAGT CCGTCCGTAT CGAGAACGCC
 851 AACCCCGCCG AGTCCTACAC CTACTCGGAC AACTCTGGCT CTTGGCAGAG
 901 CATCAAGTTC GACGGCTCCG TCGATATCTC CTCCAGCTCT TCCGTGACCT
 951 CCTCCACCAC CAGCACCGCC AGCTCCGCCA GCTCTACCTC GAGCAAGACC
1001 CCTTCCACCT CCACCCTGGC CACTTCCACC AAGGCGACTC CCACCCCGTC
1051 TGGAACCAGC TCCGGCTCTA ACTCGAGCTC CAGCGCGGAA CCTACTACCA
1101 CCGGCGGCAG CGGCAGCAGC AACACCGGCT CTGGCTCCGG CTCCGGCTCT
1151 GGCTCTGGCT CTAGCTCTAG CACGGGCTCC TCCACTAGCG CCGGAGCCTC
1201 CGCCACCCCC GAGCTCTCCC AGGGCGCCGC CGGCTCCATC AAGGGCTCGG
1251 TCACCGCCTG CGCTCTGGTG TTCGGCGCCG TCGCTGCCGT GTTGGCATTC
1301 TAA
```

Supplementary Figure S3. Consensus sequence of *CRF1* gene in *Aspergillus fumigatus*. Forty-nine strains isolated from patients were cultured. DNA was collected and *CRF1* gene was amplified. Products were then sequenced with Sanger capillary electrophoresis. Consensus sequence corresponds to the sequence found 100% conserved in 45/49 strains. Position 1110 (framed in red) corresponds to the site were a cytosine replaces a guanine in strain Af293, inducing a change of amino acid in Crf1 transcript for this strain (threonine instead of a serine for other strains).

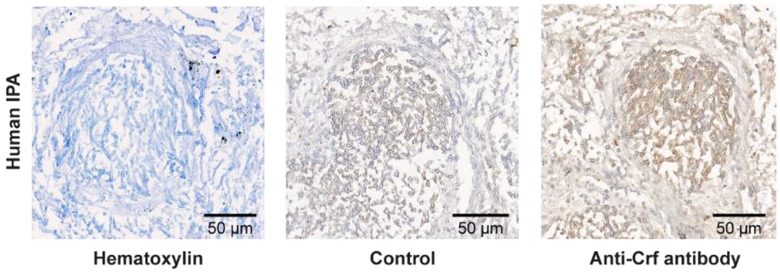

Supplementary Figure S4. Study of Crf protein in human IPA by immunohistochemistry. Identification of Crf proteins (in brown) in human lung tissues. Human lung 4 µm slides from a patient with IPA were incubated with 40 µg/mL anti-human IgA, IgG and IgM F(ab')$_2$ antibody and 8.36 µg/mL human Fc block. Anti-Crf MS112-IIB1 scFv-Fc at 2 µg/mL was used as the primary antibody (saturated or not with recombinant Crf2 protein as control), before addition of anti-human IgG coupled Biotin secondary antibody. Avidin-HRP and DAB substrate were used for development, before staining with Gill's hematoxylin. Scale bar: 50 µm. Magnification: x400.

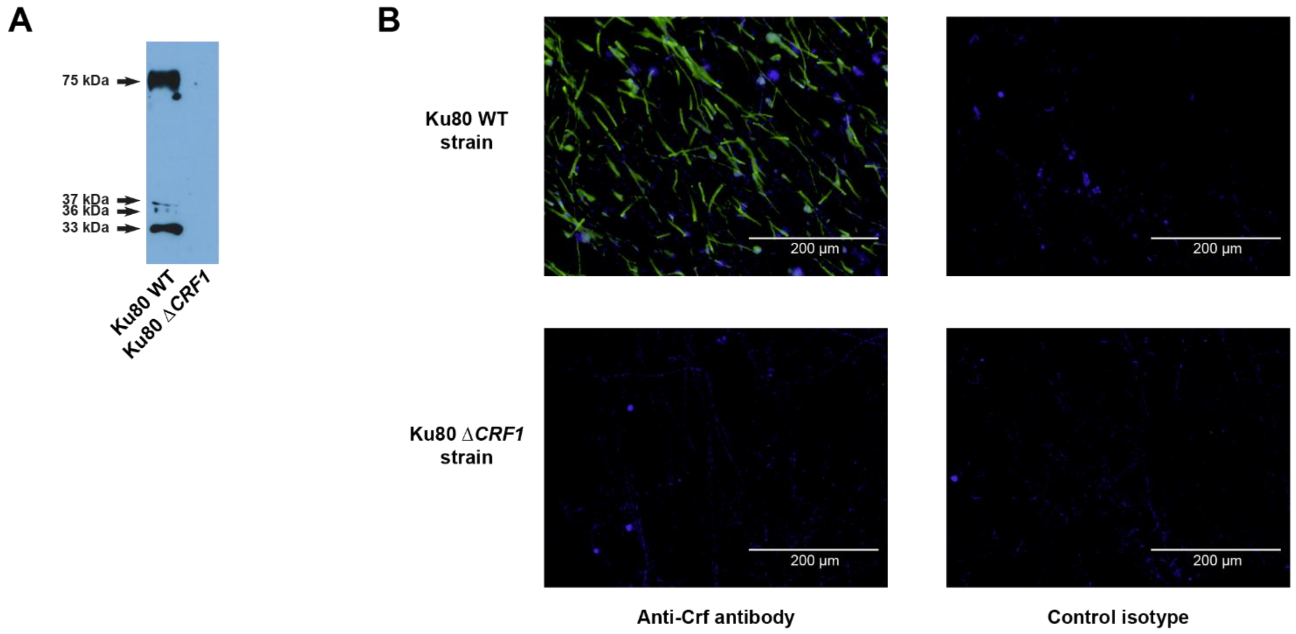

Supplementary Figure S5. Verification of *CRF1* mutant and of the absence of cross-reactivity of anti-Crf antibody. Verification of the non-expression of Crf proteins in Ku80 Δ*CRF1* strain. **(A)** Verification by Western Blot. Ku80 Wild Type strain (WT) or Ku80 Δ*CRF1* strain deleted for *CRF1* gene were incubated for 24h and their cell wall proteins were extracted. Anti-Crf MS112-IIB1 antibody at 2 µg/mL was used as primary antibody in Western Blot, before the addition of an anti-human IgG secondary antibody conjugated to HRP. **(B)** Verification by immunofluorescence. After cultivation of both strains for 24h, MS112-IIB1 IgG1 anti-Crf antibody (or Trastuzumab isotype) at 2 µg/mL were used as primary antibodies. Crf proteins (green) were identified with the addition of secondary antibody anti-human IgG Alexa Fluor 488. Cell nuclei were stained in Hoechst (in blue). Scale bar: 200 µm. Magnification: x200.

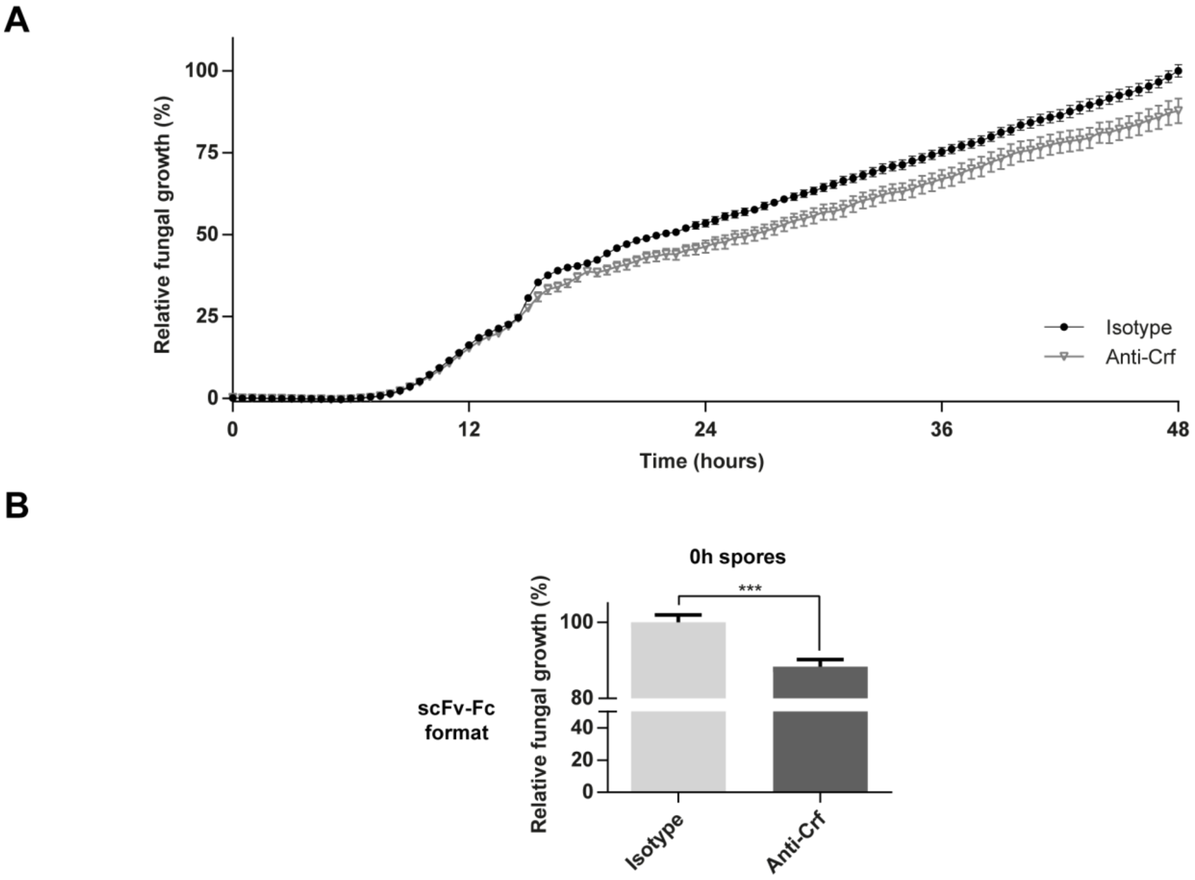

Supplementary Figure S6. Neutralization of Crf proteins on *A. fumigatus* cultures. (A) Growth kinetics of Crf+ strain in presence of anti-Crf antibodies. Anti-Crf MS112-IIB1 IgG1 antibody at 0.5 µg/mL was added to 0h spores cultured at a density of $3.5.10^4$ spores *per* well in a 96-well plate, in RPMI medium. Growth kinetics was performed at 35°C, with a reading of 530 nm absorbance (12 points per well) every 30 minutes, during 48h. Results (*n*=16) are expressed in mean ± SEM. **(B)** Neutralizing effects of anti-Crf scFv-Fc antibody on fungal growth. $3.5.10^4$ spores *per* well were seeded in a 96-well plate, in RPMI medium. scFv-Fc anti-Crf antibody or control antibody (Control) was added with the fungus at a final concentration of 0.5 µg/mL. Addition to 0h spores was studied. Absorbance at 530 nm was read after a 48h incubation at 35°C. Results (*n*=18) are expressed in mean ± SEM; Mann-Whitney statistical test was used. ***: p<0.001.

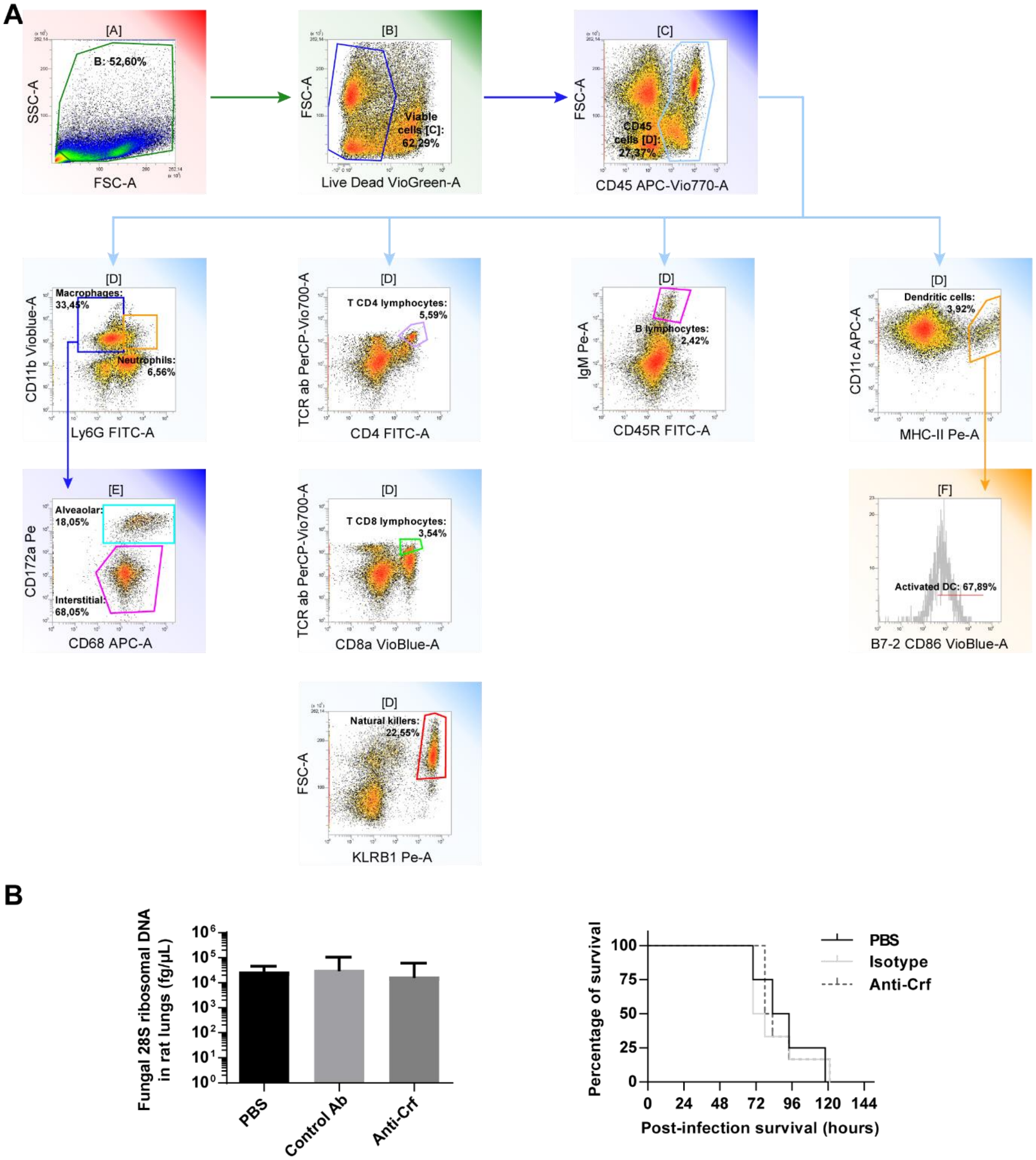

Supplementary Figure S7. Gating strategy of the different immune cells and testing of anti-Crf scFv-Fc antibody in a rat model of IPA. (A) Gating strategy for the study of immune cell populations in the rat model of aspergillosis. Neutrophils ($CD11b^{hi}$, $Ly6G^{hi}$), total macrophages ($CD11b^{hi}$, $Ly6G^{low}$), alveolar macrophages ($CD11b^{hi}$, $Ly6G^{low}$, $CD172^{hi}$, $CD68^{hi}$), interstitial macrophages ($CD11b^{hi}$, $Ly6G^{low}$, $CD172^{low}$, $CD68^{hi}$), T CD4 lymphocytes ($CD4^{+}$, TCR^{+}), T CD8 lymphocytes ($CD8a^{+}$, TCR^{+}), B lymphocytes ($CD45R^{+}$, IgM^{+}), natural killers ($KLRB1^{+}$), dendritic cells ($CD11c^{hi}$, $MHC\text{-}II^{hi}$) and activated dendritic cells ($CD11c^{hi}$, $MHC\text{-}II^{hi}$, $CD86^{hi}$) populations were studied. **(B)** Evaluation of scFv-Fc effects on the lung fungal load and on the survival of a rat model of IPA. Neutropenic rats were infected by 10^6 spores of Crf+ strain, and challenged extemporaneously either with intra-tracheal aerosolization of PBS (*n*=10), control antibody (Control Ab, *n*=12) or anti-Crf scFv-

Fc antibody (*n*=12). Antibody administered dose was 4 mg/kg. Half of rats were sacrificed after 72h to measure fungal load (left). Results are expressed in mean ± SD; Mann-Whitney statistical test was used. Other half participated to a survival study (right).
